# Changes in the flexion relaxation response induced by lumbar muscle fatigue

**DOI:** 10.1186/1471-2474-9-10

**Published:** 2008-01-24

**Authors:** Martin Descarreaux, Danik Lafond, Renaud Jeffrey-Gauthier, Hugo Centomo, Vincent Cantin

**Affiliations:** 1 Département de Chiropratique, Université du Québec à Trois-Rivières, Canada; 2 Département des Sciences de l'activité physique, Université du Québec à Trois-Rivières, Canada

## Abstract

**Background:**

The flexion relaxation phenomenon (FRP) is an interesting model to study the modulation of lumbar stability. Previous investigations have explored the effect of load, angular velocity and posture on this particular response. However, the influence of muscular fatigue on FRP parameters has not been thoroughly examined. The objective of the study is to identify the effect of erector spinae (ES) muscle fatigue and spine loading on myoelectric silence onset and cessation in healthy individuals during a flexion-extension task.

**Methods:**

Twenty healthy subjects participated in this study and performed blocks of 3 complete trunk flexions under 4 different experimental conditions: no fatigue/no load (1),  no fatigue/load (2), fatigue/no load(3), and fatigue/load (4). Fatigue was induced according to the Sorenson protocol, and electromyographic (EMG) power spectral analysis confirmed that muscular fatigue was adequate in each subject. Trunk and pelvis angles and surface EMG of the ES L2 and L5 were recorded during a flexion-extension task. Trunk flexion angle corresponding to the onset and cessation of myoelectric silence was then compared across the different experimental conditions using 2 × 2 repeated-measures ANOVA.

**Results:**

Onset of myoelectric silence during the flexion motion appeared earlier after the fatigue task. Additionally, the cessation of myoelectric silence was observed later during the extension after the fatigue task. Statistical analysis also yielded a main effect of load, indicating a persistence of ES myoelectric activity in flexion during the load condition.

**Conclusion:**

The results of this study suggest that the presence of fatigue of the ES muscles modifies the FRP. Superficial back muscle fatigue seems to induce a shift in load-sharing towards passive stabilizing structures. The loss of muscle contribution together with or without laxity in the viscoelastic tissues may have a substantial impact on post fatigue stability.

## Background

The concept of spinal stability was initially introduced by Bergmark[1] who presented a spinal stability model including 40 muscles. A few years later, Panjabi[2] developed a model of segmental spinal stability where the potential roles of passive articular structures, active muscle components and neuromuscular control were exposed. Based on biomechanical models of the spine, it was proposed that spinal stabilization should be considered the result of highly-coordinated muscular activation interacting with passive elements[3]. However, sufficient spinal stability is usually achieved by modest coordinated co-contraction of the anterior and posterior trunk muscles[3]. Spinal stability is also highly dependent on spinal load and posture [4] as well as task requirements[5]. Instability of the lumbar spine has been suggested to be both a cause and a consequence of low back pain (LBP)[3].

The flexion-relaxation phenomenon (FRP) is defined by a reduction in or silence of myoelectric activity of the lumbar erector spinae (ES) muscle observed during full trunk flexion[6]. In healthy individuals, myoelectric silence of the lumbar ES is observed in full trunk flexion. The mechanisms underlying the FRP have been proposed to represent a shift in load-sharing and spinal stabilization from active structures (ES muscles) to passive ligamentous and articular structures[7,8]. Tension in the posterior ligaments and zygapophysial joints increases during trunk flexion to a level where the active extension moment generated by the posterior muscles of the spine is no longer needed[6]. This neuromuscular response is likely to be triggered by growing mechanical load in the ligaments and disks of the lumbar spine. These articular structures are highly innervated by mechanoreceptors and nociceptors monitoring proprioceptive and noxious stimuli[9,10]. However, intramuscular electromyographic (EMG) studies have shown that deep back muscles such as the quadratus lumborum and deep lateral ES may assist spinal stabilization during fully-flexed posture [11]. McGill and Kippers [3] used an anatomically-detailed model of lumbar tissues to assess muscle and passive tissue loading during complete trunk flexion. They concluded that even without significant activity in the superficial ES muscle during the FRP, the passive components of these muscles are likely to contribute to spinal stability.

Previous investigations have explored the effects of load, angular trunk velocity and posture on FRP parameters. Gupta[7] observed that loading of the lumbar spine by the addition of weights prolonged the myoelectric activity of the ES and increased the range of motion. However, another study failed to obtain a significant effect of loading on FRP parameters, but showed that increasing trunk movement velocity significantly delayed the flexion angle at which ES myoelectric silence appears[12]. O'Sullivan et al.[13] examined the FRP in superficial lumbar multifidus and transverse fibers of the internal oblique muscle during slump sitting, and found inconsistent EMG patterns in the thoracic ES. Solomonow et al.[14] studied the FRP after a static lumbar flexion and noted a decreased EMG silent period. The authors proposed that the muscles may compensate for the loss of tension in the lumbar viscoelastic tissues. Finally, Olson and Solomonow[15] studied the effect of repeated cyclic lumbar flexion and concluded that modifications in the EMG patterns along cycles may be caused by increasing muscular fatigue. Their results indicated that ES myoelectric silence appeared sooner during flexion and later during spine extension. They suggested that the earlier cessation of EMG activity observed during flexion and delayed activation of the trunk extensors during extension may be caused by muscular fatigue.

Despite these preliminary results, the effects of muscular fatigue on FRP parameters have not been thoroughly studied. Therefore, the objective of the present experiment was to quantify the influence of lumbar ES muscle fatigue and the possible interaction with spine loading on myoelectric silence during the FRP in healthy individuals. Since the lumbar ES muscle is believed to play an important role in lumbar stability, it is hypothesized that its fatigue will precipitate load transfer to passive articular structures during the FRP. Such transfer to passive load structure should lead to an earlier onset of myoelectric silence during the flexion motion and a delayed FRP cessation during trunk extension.

## Methods

### Participants

Twenty healthy adult subjects, 9 men and 11 women, with no history of LBP participated in this study. All participants gave their informed, written consent according to the protocol approved by the Université du Québec à Trois-Rivières (Canada) Ethics Committee. Subjects with present or past LBP or thoracic pain, spinal trauma or surgery were excluded from the experiment. Subject characteristics are presented in Table 1.

**Table 1:** Subject characteristics

Age (years)	23.9 ± 4.0
Weight (kg)	64.1 ± 13.1
Height (m)	1.70 ± 0.10
Body mass index (kg/m^2^)	22.2 ± 3.2
Time until exhaustion (Sorensen protocol)	138 sec ± 37 sec

Data are mean ± standard deviation

### Experimental protocol

Subjects were tested during one session of approximately 60 minutes in the laboratory. Instructions followed by a demonstration of the flexion-extension task were given to the study subjects prior to the experimental trials. The subjects stood with their arms by their side and were required to bend forward as far as possible during a 5-s movement period (flexion phase). Then, they were instructed to hold the fully flexed position for a 3-s period. The extension phase lasted 5-s during the subjects' return to the initial upright standing position. An auditory cue served to standardize the movement duration throughout the different movement phases. The phase durations were not monitored online and none of the trials was rejected due velocity variations. Sufficient practice was allowed to ensure that the study subjects performed the task correctly prior to data acquisition.

The subjects performed 3 trials of the FRP task under 4 different experimental conditions: (1) no fatigue/no load, (2) no fatigue/load, (3) fatigue/no load, and (4) fatigue/load. The "non-fatigue" conditions (1), (2) were always presented before the "fatigue" conditions (3), (4). However, the "load" condition was randomized across subjects. For the loading condition, a 12-kg barbell was held with the elbows flexed at a 90° angle and upper arms hanging along the trunk. To assure constant upper limb posture between the loaded and unloaded conditions, the subjects were asked to hold a light plastic panel replicating the barbell's shape. Lumbar musculature fatigue was induced according to the Sorenson fatigue protocol. Briefly, each subject lay prone with the iliac crest aligned with the edge of the table[16] and the arms crossed on the upper trunk. The lower body was fixed to the table by straps at the pelvis, knees, and ankles to limit lower limb muscle activation. The study subjects were asked and verbally encouraged isometrically to maintain the horizontal, unsupported position of the trunk as long as possible.

### Instrumentation

Kinematic data were collected by a motion analysis system (Optotrak Certus, Northern Digital, Waterloo, Canada). Light-emitting diodes were positioned on the left side of each subject on the following anatomical landmarks: a) acromion, b) middle of the iliac crest, c) greater trochanter, d) lateral part of the knee, and e) lateral malleolus. Kinematics data were collected at 100 Hz and low-pass filtered by a dual-pass, fourth-order Butterworth filter with a cut-off frequency at 5 Hz.

Surface EMG data were collected by bipolar disposable surface Ag-AgCl electrodes applied bilaterally at the ES at the L2 level and L5 level. According to Merletti[17], electrodes were positioned in regard to muscle fibre direction, and skin impedance was reduced by: 1) shaving excess body hair, if necessary, 2) gently abrading the skin with a fine-grade sandpaper (3 M Red Dot Trace Prep) and wiping the skin with alcohol swabs. EMG activity was recorded with a Bortec biomedical acquisition system (Model AMT-8, common mode rejection ratio of 115 dB at 60 Hz, input impedance of 10 GΩ) and sampled at 900 Hz, using a 12-bit A/D converter (PCI 6024E, National Instruments, Austin, TX). EMG data were digitally filtered by a 10- to 450-Hz bandpass, zero-lag, fourth-order Butterworth filter. The data were collected by LabView and processed by MatLab.

### Data analyses

Preliminary analyses showed no gender effects on the main variables and therefore all participants' data were pooled together. Lumbar muscle fatigue was induced using the Sorenson protocol. The subjects were instructed to maintain the unsupported body (head, arms and trunk) in a horizontal position relative to the ground as long as they could, with arms crossed at the chest. Muscle fatigue during the Sorenson protocol was assessed through power spectral analysis of the EMG data (fast fourrier transform). The rate of decline of median frequency (MedF) with time and the concomitant rate of increase in EMG amplitude (root mean square: RMS) with time were calculated to confirm that lumbar muscular fatigue was induced correctly[18,19]. More precisely, RMS and MedF were calculated from equally-spaced windows of 250 ms every 3 s during the first 60s the Sorenson test. To estimate the rate of muscle fatigue, we applied least-squares linear regression analysis to calculate the slope of MedF over time (MedF/time slope) and the RMS over time (RMS/time slope).

**Figure 1:**
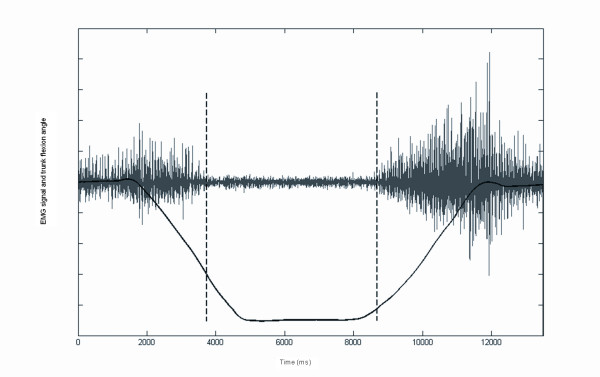
Scaled EMG raw signal and total trunk angle from a subject performing a FRP task in a no load, no fatigue condition.

The EMG data were full wave-rectified and normalized to maximal muscle (%MVC) activity obtained during maximal voluntary isometric contraction. During the MVC protocol, the subjects performed, while standing in a dynamometer (LIDO, Loredan biomedical, Davis, CA) several static trunk extension efforts. The feet were secured to prevent slipping and the hip was strapped firmly on the dynamometer. The experimental upright posture was 20 degrees of the hip and trunk flexions. To minimize lateral bending moment and axial trunk rotation efforts, subjects were instructed to produce L5/S1 extension in the sagittal plane only. They were asked to perform the following sequence: two to four submaximal contractions to become familiar with the task and three maximal voluntary contractions (MVC). These trials were separated by a 60 s rest period. During the MVC, verbal encouragement was provided to each subject to ensure maximal effort. The rectified EMG signals and kinematics data were then plotted to determine total trunk angle corresponding to EMG cessation during the flexion phase and the total trunk angle of EMG onset during the extension phase. EMG cessation and onset were quantified by visual inspection of the rectified EMG signal (Figure 1). The flexion relaxation ratio (FRR) was also calculated by dividing RMS activity measured during movement, either in the flexion (FRR_F_) or extension (FRR_E_) phases, by the RMS while relaxed (full flexion phase).

### Statistical analyses

Total flexion angle corresponding to the onset and cessation of myoelectric silence, the knee angle and the FRR as well as the mean duration for the flexion and extension phases were compared across the different experimental conditions by 2 × 2 (Load × Fatigue) repeated-measures ANOVA. To determine possible gender main effect or interactions genders, a repeated-measures ANOVA was performed for each variable. No significant differences were found, so data from both sexes were pooled. Statistical significance level was set to p < 0.05 for all analyses.

## Results

**Figure 2:**
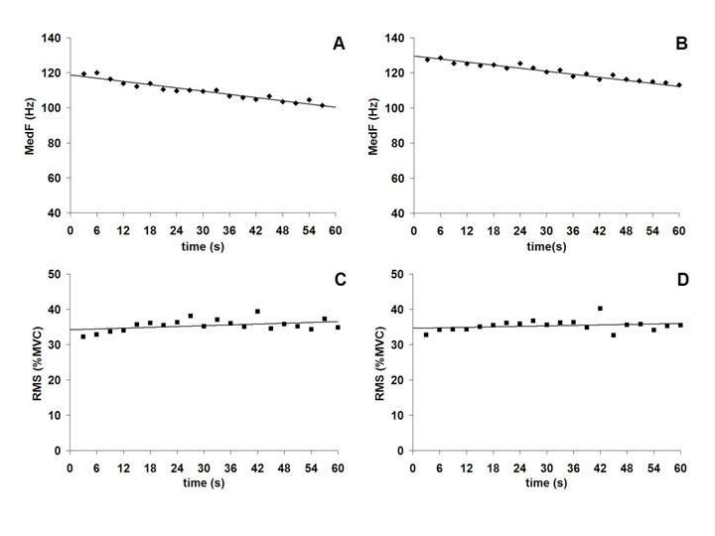
MedF/time and RMS/time slopes of the ES-L2 (A, C) and the ES-L5 (B, D) during the Sorensen fatigue protocol. The results are the average of all subjects.

EMG fatigue indices (RMS/time, MedF/time) are presented in Figure 2 for both L5 and L2 ES levels. The mean time until exhaustion (Sorensen protocol) is presented in Table 1. For all subjects, a rate of decline in MedF/time (-0.69 ± .36 Hz at L2; -0.81 ± .40 Hz at L5) and a rate of increase in RMS/time (0.02%MVC/s at L2; 0.03%MVC/s at L5) were observed, indicating that muscular fatigue was induced prior to the FRP tasks. Although a metronome was used to standardize movement velocity and guide the participants throughout the different movement phases, we also computed movement speed and found no significant difference in the duration of FRP phases between conditions. The experimental conditions had no significant effect on the duration trunk movement (*P *> 0.05). Mean durations, for the flexion and extension phases respectively, were 4.00 ± 0.14 s and 3.99 ± 0.18 s for condition [1], 4.06 ± 0.10 s and 4.21 ± 0.21 s for condition [2], 3.95 ± 0.14 s and 4.13 ± 0.15 s for condition [3], and 3.90 ± 0.15 s and 3.92 ± 0.15 s for condition [4]. Furthermore, the experimental conditions had no significant effect on maximum knee angle during the FRP task, confirming that all trials were performed similarly across conditions ([1] 168.7 ± 1.2, [2] 168.9 ± 1.1, [3] 167.9 ± 1.4, [4] 167.5 ± 1.2; F_1,19 _= 0.331; *P *> 0.05).

**Figure 3:**
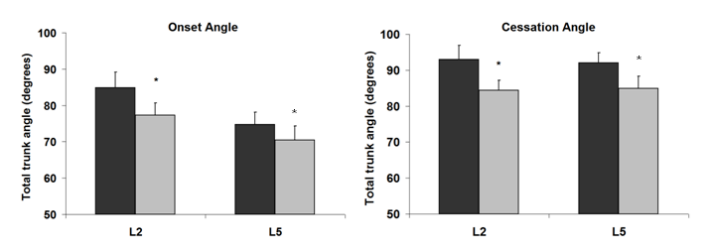
Effect of fatigue induced at each muscle level (no fatigue = black; fatigue = grey) on total trunk angle at FRP onset and at FRP cessation. *P < 0.05.

**Figure 4:**
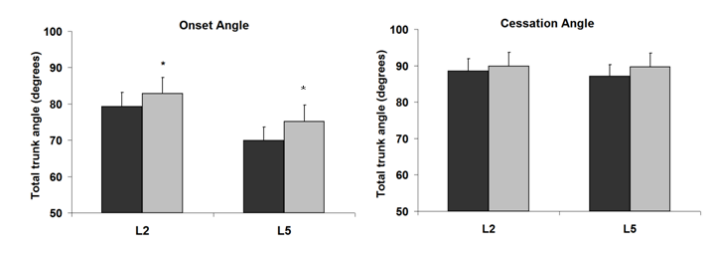
Effect of loading at each muscle level on total trunk angle at FRP onset and at FRP cessation. *P < 0.05. (no fatigue = black; fatigue = grey).

**Figure 5:**
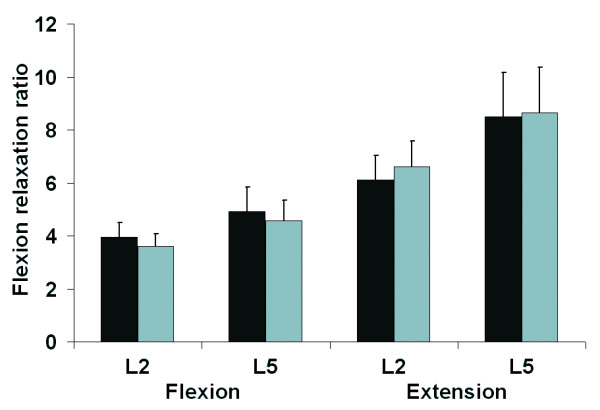
Effect of loading on the FRR during flexion movement and extension movement for each ES level (no load = black; load = grey).

**Figure 6:**
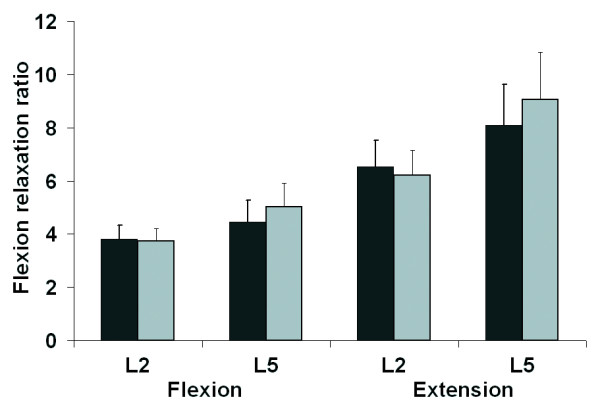
Effect of fatigue on the FRR during flexion movement and extension movement for each ES level (no fatigue = black; fatigue = grey).

We found a significant effect of muscular fatigue on both FRP cessation and FRP onset angles for all muscles (Figure 3). The angle corresponding to the onset of myoelectric silence was significantly reduced after the fatigue task for both the L2 level (84.9 ± 4.3 vs 77.3 ± 3.4; F_1,16 _= 17.2; *P *< 0.01) and the L5 level (74.7 ± 4.8 vs 70.4 ± 3.9; F_1,15 _= 11.7; *P *< 0.01). Additionally, the angle corresponding to the cessation of myoelectric silence was significantly decreased after the fatigue task for both the L2 level (93.0 ± 3.9 vs 84.4 ± 2.8; F_1,16 _= 11.4; *P *< 0.01) and the L5 level (92.0 ± 4.1 vs 84.9 ± 3.4; F_1,16 _= 15.1; *P *< 0.01). These results indicated that lumbar muscle fatigue produced a shift on the FRP, appearing sooner during flexion movement and later during extension movement. There was also a significant increase in FRP onset angle during loading conditions (Figure 4). However, loading conditions did not affect FRP cessation angle at both the L2 and L5 levels (Figure 4). Figures 5 and 6 illustrate that there was no significant influence of loading or fatigue conditions on the FRR_F _and FRR_E _at both L2 and L5 levels (*P *> 0.05).

## Discussion

### Fatigue effect

The results of the present study indicate that muscular fatigue of the lumbar ES modulates the FRP. In healthy subjects, fatigue of the lumbar ES, with or without an additional load, increases the myolectric silence period during a flexion-extension task. Olson et al.[15] reported similar data in a recent article, and observed an extended EMG silent period with prolonged cyclic lumbar flexion. Similarly to our findings, this augmented silent period was produced from both a reduction in onset angle of the FRP and a decreased FRP cessation angle. These authors hypothesized that the observed changes in FRP parameters were caused by muscular fatigue occurring throughout cyclic lumbar flexion. Unfortunately, muscle fatigue was not an independent variable in their study design. In fact, cyclic lumbar flexion may induce muscular fatigue of the lumbar ES muscle, but it will also generate creep in the passive viscoelastic structures of the spine, therefore modifying the spine mechanoreceptor threshold. Dickey et al. [20] reported opposite results in a similar task and observed a shortened silent period after repeated flexion-extension. On the other hand, Solomonow et al.[14,21,22] showed that creep developed in the lumbar viscoelastic tissues during a period of cyclic or static flexion significantly reduces stabilizing capacity of passive articular tissues. Our results, together with the recently published results clearly indicate that the loss of muscle contribution together with or without laxity in the viscoelastic tissues may have a substantial impact on post fatigue stability due to static or cyclic loading. To date, no study has addressed the interrelated or combined neuromuscular influences of viscoelasticity property changes and lumbar muscular fatigue on the FRP.

To independently assess the effect of fatigue on the FRP, lumbar ES fatigue was evoked according to the Sorenson protocol. Previous reports indicated that the Sorenson protocol was one of the most effective tests of lumbar muscle fatigue induction[23,24]. Our results revealed that lumbar muscle fatigue was appropriately produced prior to the FRP tasks. An isometric test to induce fatigue prevented the possible effect of creep development in the spinal ligaments and, therefore, enabled us to solely assess the effect of fatigue on the FRP. Sarti et al. [12] studied the effect of movement speed on the FRP parameters. Their results showed that increased speed of movement significantly delayed the appearance of electrical silence in the range of flexion. They suggested that the viscoelastic properties of spine tissues might have triggered different responses at different movement speeds, therefore modifying FRP onset angle. In the present experiment, flexion and extension movement durations were constant throughout the four experimental conditions, and the observed changes during the fatigue and loading conditions could not be attributed to unconscious modification in speed execution of the flexion-extension task.

The lumbar ES muscles are believed to be involved in stabilization of the lumbar spine in various tasks [3]. It is possible that these muscles, in a state of fatigue, are not able to provide sufficient stabilization to the vertebral units, transferring load-sharing to passive structures earlier in trunk flexion. It has also been suggested in the literature that deep back muscles such as the quadratus lumborum and deep lateral ES are involved in creating extensor moment during trunk flexion[11]. The decreased angle corresponding to the cessation of myoelectric silence during fatigue observed in our study might reflect heightened activation of the deep muscles compensating for superficial lumbar ES fatigue. Considering that the subjects held a fully-flexed posture for 3 s, one could argue that the deep back muscles initiated trunk extension in the fatigue condition whereas the superficial muscles initiated extension in the non-fatigue condition. Research showed that the quadratus lumborum acts as an agonist of the extensors in extending the spine[3]. Intramuscular recording would be necessary to confirm possible muscle synergies between the superficial and deep trunk muscles during loading and fatigue conditions. Additionally, investigating the effect of fatigue in other trunk-stabilizing muscle groups, such as the abdominals[25] and hip extensors [26-28], could shed light on the load transfer mechanisms involved during trunk flexion and extension. The functional role of the hip extensor muscles including the gluteus muscles and the hamstring muscles have been extensively studied and they seem to be actively involved in lower back stabilization as well as in lumbopelvic rhythm[28,29]. For instance, van Wingerden et al. (2004) showed that the biceps femoris and gluteus maximus muscles can increase sacroiliac joint stabilization through their specific and massive attachments to the sacrotuberous ligament. Their results also indicated that the erector spinae and hip extensor muscles clearly interact to provide adequate lumbopelvic stabilization and it seems that hip extensor muscles and erector spinae are both anatomically and functionally linked during the trunk flexion task.

### Load effect

The addition of a load anterior to the trunk modified the FRP response. FRP onset angle was increased in load conditions, whereas no significant effect of load was noted for FRP cessation angle. Several authors have reported a similar effect of load positioned either anteriorly or posteriorly to the trunk [7,20]. Such a decrease in the EMG silence period during flexion reflects the need for additional muscular contraction to counteract the increased flexion moment generated by the load. On the other hand, Sarti et al. [12] recorded a significant effect of speed on FRP onset angle, but did not observe any significant influence of load.

### Clinical implications

Subjects with chronic LBP often lack the FRP response by persistent ES muscle activity in full flexion [30-34], and the absence of the myoelectric silence phase can be observed even in chronic LBP subjects who are in a symptom-free period[35]. The increased myoelectric activity seen in LBP subjects during the FRP has been attributed to several factors, such as muscle spasm, reduced range of motion, exaggerated stretch reflexes, effort to protect damaged passive structures or response to local instability caused by injured spinal structures [6,16,33]. Such a transient response may help chronic LBP patients to maintain satisfactory functional status. According to biomechanical models of the lumbar spine, spinal stability may be compromise due to insufficient muscle force and inappropriate neuromuscular activation[3]. Therefore, muscular fatigue of the lumbar ES may temporarily reduce spinal stability during full flexion and subsequently put previously-injured structures at risk.

## Conclusion

Our results, together with recent data published by Olson et al.[36] who studied the influence of gravitational loading orientation on the anterior and posterior lumbar muscles during trunk flexion-extension performed from supine and standing positions indicate that external load is a major modulator of the FRP. Interactions between chronic pain and muscular fatigue and their effect on the FRP need to be studied. The results of such investigations could greatly impact the management of chronic LBP rehabilitation programs.

## Competing interests

The author(s) declare that they have no competing interests.

## Authors' contributions

MD and DL participated in the design of the study, experimentation, data analysis and writing of the manuscript. RJG participated in the experimentation and data analysis. Finally, HC and VC helped in the data analysis, statistical analysis and draft of the manuscript. All authors read and approved the final manuscript.

## Pre-publication history

The pre-publication history for this paper can be accessed here:


